# Binding-Folding Induced Regulation of AF1 Transactivation Domain of the Glucocorticoid Receptor by a Cofactor That Binds to Its DNA Binding Domain

**DOI:** 10.1371/journal.pone.0025875

**Published:** 2011-10-07

**Authors:** Anna S. Garza, Shagufta H. Khan, Carmen M. Moure, Dean P. Edwards, Raj Kumar

**Affiliations:** 1 Department of Basic Sciences, The Commonwealth Medical College, Scranton, Pennsylvania, United States of America; 2 Department of Internal Medicine, University of Texas Medical Branch, Galveston, Texas, United States of America; 3 Department of Molecular and Cellular Biology and Pathology, Baylor College of Medicine, Houston, Texas, United States of America; Ohio State University, United States of America

## Abstract

Intrinsically disordered (ID) regions of proteins commonly exist within transcription factors, including the N-terminal domain (NTD) of steroid hormone receptors (SHRs) that possesses a powerful activation function, AF1 region. The mechanisms by which SHRs pass signals from a steroid hormone to control gene expression remain a central unresolved problem. The role of N-terminal activation function AF1, which exists in an intrinsically disordered (ID) conformation, in this process is of immense importance. It is hypothesized that under physiological conditions, ID AF1 undergoes disorder/order transition via inter- and intra-molecular communications, which allows AF1 surfaces to interact with specific co-regulatory proteins, critical for the final outcome of target gene expression regulated by SHRs. However, the means by which AF1 acquires functionally folded conformations is not well understood. In this study, we tested whether binding of jun dimerization protein 2 (JDP2) within the DNA binding domain (DBD) of the glucocorticoid receptor (GR) leads to acquisition of functionally active structure in its AF1/NTD. Our results show that signals mediated from GR DBD:JDP2 interactions in a two domain GR fragment, consisting of the entire NTD and little beyond DBD, significantly increased secondary/tertiary structure formation in the NTD/AF1. This increased structure formation facilitated AF1’s interaction with specific co-regulatory proteins and subsequent glucocorticoid response element-mediated AF1 promoter:reporter activity. These results support the hypothesis that inter- and intra-molecular signals give a functionally active structure(s) to the GR AF1, which is important for its transcriptional activity.

## Introduction

The glucocorticoid receptor (GR) is a ligand-activated intracellular transcription factor with the domain structural arrangement typical of the nuclear hormone receptors (NHRs) superfamily [1], [2], [3], [4], [5]. Like other NHRs, GR regulates transcription of target genes by binding DNA at specific glucocorticoid response elements (GREs) and by interacting with other coregulatory proteins [6], [7], [8], [9]. However, precisely how transcription is regulated by the GR is largely unknown. There are at least two defined transcription activation functions (AFs) that provide protein interaction surfaces for coregulatory proteins: AF1 in the N-terminal domain (NTD) and a conserved ligand-dependent AF2 in the ligand binding domain (LBD) [10], [11], [12], [13], [14], [15]. A major challenge is to understand the role of these AFs. Availability of crystal structure of the LBD has immensely helped in our understanding about AF2 functions [16]. The lack of knowledge is most marked concerning the way in which AF1 (that controls majority of GR’s transcriptional activity) functions [17], [18], [19]. This activity appears to be cell/coactivator-dependent [20], [21], [22], [23], [24]. Because AF1 exists in an intrinsically disordered (ID) conformation, understanding its structure:function relationship has languished. It is known that AF1 interacts with other co-regulatory proteins, and the available data strongly suggest that conditional folding of AF1 is the key for many of these interactions and subsequent transcriptional activity [17]. How and what kind of functionally folded conformation AF1 adopts is an important question. Recent studies have shown that several ID regions/domains undergo a disorder/order transition upon direct interaction with their target proteins including GR AF1 [25], [26], [27]. However, it is not known whether a binding partner protein that interacts with the GR outside AF1 domain also leads to imposition of a functionally active conformation in AF1.

Jun dimerization protein-2 (JDP2) is a small bZIP protein that contains the leucine zipper and the basic amino acid DNA binding domain common to AP-1 factors but lacking an N-terminal activation domain [28], [29], [30]. JDP2 is known to interact with the DBD and the carboxyl terminal extension (CTE; a non-conserved region on the immediate C-terminal side of the conserved 2^nd^ zinc finger of the core DBD) of human progesterone receptor (PR) and enhance PR’s transcriptional activity, independent of AF2 and p160 coactivators [31], [32], [33], [34]. In the present study, we sought to determine whether JDP2 can interact with the GR and modulate its transcriptional activity. We show that JDP2 interacts with GR's DBD/CTE region and induces a compact structure in NTD/AF1 in a manner that facilitates AF1’s interaction with specific co-regulatory proteins and correlates with the stimulation of AF1-mediated transcriptional activity.

## Materials and Methods

### Plasmids

The pGRE_SEAP vector (BD Biosciences, Palo Alto, CA) contains three copies of a GRE consensus sequence in tandem, fused to a TATA-like promoter (*P*_TAL_) upstream from the reporter gene for secreted alkaline phosphatase (SEAP). GR500 encodes amino acids 1–500 of the human GR, plus a five-residue nonspecific extension [20], [21], [35]. TBP was cloned into the pcDNA3.1(+) expression vector (Invitrogen, Carlsbad, CA), and into pEYFP-C1 (BD Biosciences). Plasmids for pYFP-CBP and p-YFP-SRC-1 constructs were kindly provided by Dr. M. Mancini, Baylor College of Medicine. Construction of pCR3.1-JDP2 mammalian plasmid has been previously described (32). The CFP-YFP fusion protein was generated as described [36], [37].

#### Expression and Purification

Construction, expression, and purification of the GR500, GR465*, AF1, DBD, TBP_C_, and JDP2 have been described [21], [25], [32], [33], [36]. Protein purity (95% or more) was analyzed by Coomassie blue staining.

#### GST-pull down assay

Purified GST-500; GST-465*, GST-AF1, GST-DBD, or GST protein was immobilized on glutathione-Sepharose beads. Purified JDP-2 or TBP_C_ were added to the GST-bound beads, and the mixture was further incubated for 2 h. Any unbound protein was washed thoroughly. To the washed beads, SDS-PAGE sample buffer was added, and the sample boiled for 5 min in a water bath. Each sample was then run on a SDS-PAGE gel and visualized by Coomassie Blue R-250 staining.

#### Circular Dichroism (CD) Spectroscopy

CD spectra of GR500, JDP2, and GR500:JDP2 mixtures were recorded at 22°C on a Jasco-815 spectropolarimeter by using a 0.1-cm quartz cell, with a bandwidth of 1.0 nm and a scan step of 0.5 nm. Similar approaches were applied to other GR fragments (AF1 or DBD). Each spectrum was corrected for the contribution of solute concentrations, and is a result of five spectra accumulated, averaged, and smoothed.

#### Cell Culture, Transient Transfection

CV-1 monkey kidney epithelial cells (American Type Culture Collection) were grown at 37°C in MEM with Earle’s salts (Invitrogen) supplemented with 10% (vol/vol) fetal bovine serum (Atlanta Biologicals, Norcross, GA). CV-1 cells were plated on a 24-well plate (500 µl/well) 1 day before the transfection and transfected using Lipofectamine 2000 (Invitrogen) according to the manufacturer’s protocol. Transfected cells were maintained at 37°C in 5% CO_2_/95% during the experiments.

#### Fluorescence Microscopy and FRET Analysis

CV-1 cells were grown on a tissue culture dish with integrated slide (Matec) for 1 day before transfection. Several control experiments were included. Independent CFP and YFP-expressing constructs were tested for FRET as negative control. As a positive control, a CFP-YFP construct that linked CFP-YFP by eight amino acids was co-expressed. CV-1 cells were cotransfected with 1 µg of pGRE-SEAP reporter and 3 µg of pECFP-YFP (positive control), 1.5 µg of pECFP-C1, and/or 1.5 µg of pEYFP-C1 (negative control). To test the dependence of FRET on AF1 in the GR, cells were cotransfected with 1.5 µg of pEYFP-TBP, -CBP, or –SRC-1 and 1 µg of pGRE-SEAP. Pairs of pGRE-SEAP received 1.5 µg of pECFP-GR500. Cells were washed 24 h later twice with isotonic pH 7.4 PBS, fixed with 4% formaldehyde/PBS for 10 min and washed twice with PBS. Cells were visualized using a Zeiss LSM-510 META confocal microscope (Carl Zeiss, Thornwood, NY) with a Plan-Apochromat 63×1.4 oil-immersion objective and 6.1 Amp Argon laser. Pre- and post-bleach (PB) images were collected at 12-bits resolution on two channels: 458 nm for CFP and 514 nm for YFP. Five images were taken, two before and three after the PB, with 20-sec intervals. To assure more than 90% PB, an arbitrarily selected region of interest, containing examples of both nuclear and cytoplasmic compartments, was irradiated with the 100% intensity laser line at 514 nm at 200–2000 iteration. Increased CFP (donor) fluorescence intensity upon YFP (acceptor) was indicative of positive FRET and its efficiencies (FE) were calculated by the equation: FE% = (I_DA_−I_DB_)/(I_DA_)×100. Where, I_DA_ is donor intensity after PB (extracted from image 2 of time series) corrected for background and fractional PB; I_DB_ is donor intensity before PB background corrected (estimated from image 3 of the PB time series). Images that showed any focal plane drift were eliminated. In addition, we tested CFP, CFP-GR500, YFP, and YFP-TBP alone each time to account for any bleed-through and background FRET as recommended.

#### Reporter Gene Assays

We employed the secreted alkaline phosphatase (SEAP) reporter system due to its high signal-to-noise ratio and quantifiable transcriptional activity without the need for cell disruption. CV-1 cells were cotransfected as described above with 0.13 µg of pGRE_SEAP reporter vector, 0.13 µg of pECFP-GR500, and 0.5µg of pcDNA3.1-TBP, pRSLV-CBP, or SRC-1. The total amount of DNA added was kept fixed at 0.8 µg by addition of empty pECFP vector. Medium (25 µl) was collected 27 h later and tested for the presence of SEAP (Great EscAPe SEAP Detection Kit; BD Biosciences) according to the manufacturer’s protocol. Data from different experiments were normalized to GR500 activity.

## Results

### JDP2 interacts with the GR through its DBD

**Figure 1:**
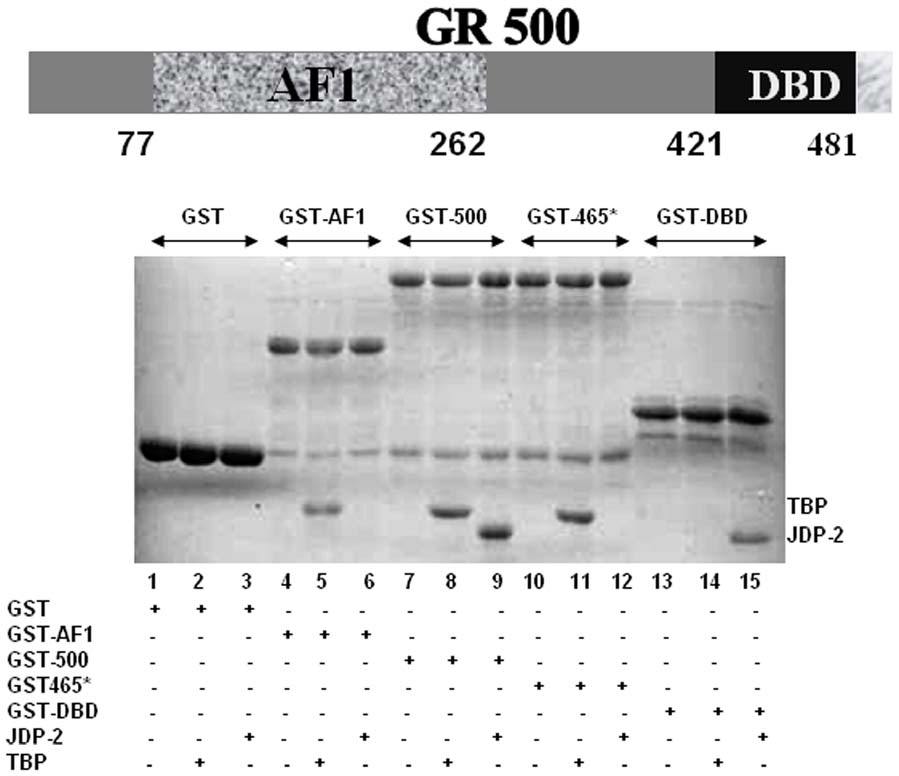
JDP2 binds to the C-terminal part of the GR DBD. A) A topological diagram of the GR500 fragment showing NTD (a.a. 1–420), AF1 (77–262), and DBD (421–481). B) Coomassie-stained SDS-PAGE gel showing the patterns of interactions between JDP2 or TBP_C_ and various fragments of the GR (indicated on the top). GST-pull down assay was performed to determine these *in vitro* interactions using purified recombinant protein in each case.

In the present study, we analyzed binding of JDP2 to GR and mapped the region of interaction within GR by *in vitro* GST pull-down assays. GST-GR500 (Figure 1A) was immobilized onto glutathione Sepharose-4B resin, incubated with JDP2 and bound JDP2 was detected by Coomassie blue stained SDS gels. Figure 1B shows that GR500 interacted efficiently with JDP2 (lane 9) but not with the free GST control (lane 3). To map the region of GR required for interaction with JDP2, various fragments of GR were used in pull-down assays. A construct containing the AF1 domain (amino acids 77–262 of human GR; AF1) did not interact with JDP2 (lane 6), whereas a GR fragment consisting of amino acids 398–500 (DBD) did interact with JDP2 (lane 15). These results suggest that JDP2 interacts with the DBD of the GR**.** To further map the region of the DBD, we used a fragment of the GR, which consists of amino acids 1–465 of the human GR plus 21 extra amino acids (GR465*; that is random in nature and does not match GR sequences beyond amino acid 465 [21]. Our results show that JDP2 fails to interact with this GR fragment (lane 12) suggesting that binding of JDP2 to GR requires amino acid sequences 465–500. This region encompasses part of the 2^nd^ zinc finger in the DBD core plus part of the CTE and thus corresponds with the same region of PR that interacts with JDP2 [33]. We have earlier shown that TATA box binding protein (TBP_C_) interacts with the AF1 domain of the GR [25]. As a control, we used TBP_C_ to demonstrate that our data showing interactions between the GR DBD and JDP2 is specific. We determined the binding of TBP_C_ to each of the fragments used for JDP2 interactions. As expected, TBP does not show any interaction with GST alone (lane 2) whereas it interacts with each of the GR fragments that possess AF1 domain (lane 5 for AF1; lane 8 for GR500; lane 11 for GR465*). When we used GST398-500 (DBD) alone, we could not detect any interaction of TBP_C_ with this GR fragment (lane 14). Taken together, our data clearly indicate that JDP2 interaction requires the part of the core DBD and CTE region between amino acids 465–500, in contrast with TBP that interacts directly with AF1/NTD.

### JDP2 binding to DBD in a two-domain GR fragment containing the NTD and DBD (GR500) induces a compact structure in NTD/AF1 domain

**Figure 2:**
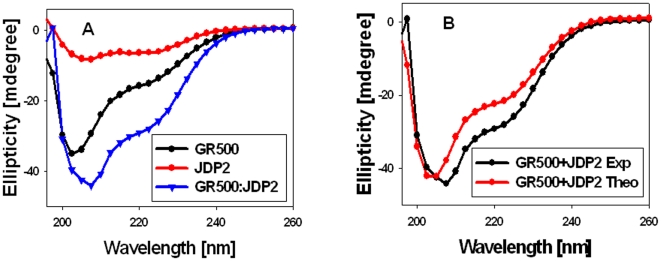
JDP2 binding to the GR DBD induces secondary structure in ID NTD/AF1 domain. A) Far-UV CD spectra of recombinant GR500, JDP2, and GR500:JDP2 mixture. B) Far-UV CD spectra of GR500:JDP2 mixture (experimental), and additive of GR500+JDP2 (theoretical sum of GR500 plus JDP2). Each spectrum presented is the average of five spectra recorded, corrected for the contribution of the buffer, and smoothed.

**Figure 3:**
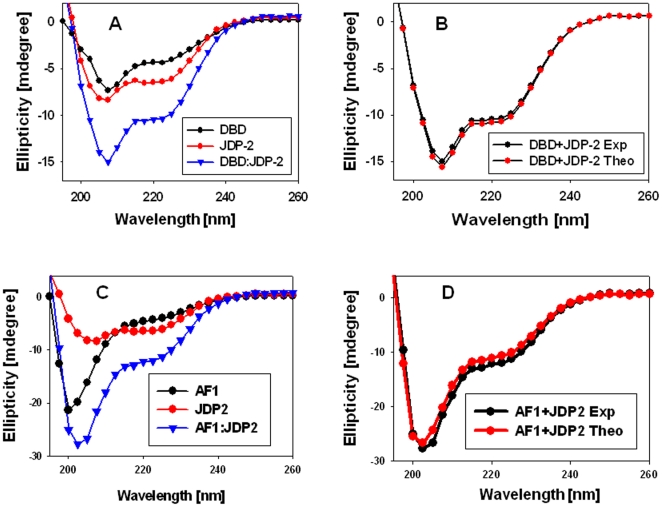
JDP2 binding to the GR DBD fails to induce any significant structural changes in DBD. A) Far-UV CD spectra of recombinant DBD (a.a. 398–500), JDP2, and DBD:JDP2 mixture. B) Far-UV CD spectra of DBD:JDP2 mixture (experimental), and additive of DBD+JDP2 (theoretical sum of DBD plus JDP2). Each spectrum presented is the average of five spectra recorded, corrected for the contribution of the buffer, and smoothed. C) Far-UV CD spectra of recombinant AF1 (a.a. 77–262), JDP2, and AF1:JDP2 mixture. D) Far-UV CD spectra of AF1:JDP2 mixture (experimental), and additive of AF1+JDP2 (theoretical sum of AF1 plus JDP2). Each spectrum presented is the average of five spectra recorded, corrected for the contribution of the buffer, and smoothed.

We next determined whether JDP2 interaction with the GR DBD induces a compact structure in otherwise ID NTD/AF1 domain. We first analyzed far-UV CD spectra of JDP2, GR500, and a GR500:JDP2 mixture (1∶2 molar ratios), detecting ellipticity for all three in a wavelength range of 190 nm and 260 nm (Figure 2A). As expected, JDP2 spectrum shows two major negative peaks at 208 nm and 222 nm, indicating that it adopts a primarily α-helical conformation in solution [32], [38]. The GR500 fragment exhibits characteristics of polypeptides that contain a large amount of random coil with some underlying helical content [35]. When the spectrum of a mixture of GR500 and JDP2 is compared with either alone, we found that the protein complex (GR500:JDP2) has significantly higher secondary structural elements than either protein alone (Figure 2A). CD analysis of a mixture of two interacting proteins that do not undergo a conformational change should yield a spectrum that should be more or less super-imposable in comparison to the theoretical sum of the spectra of the two proteins measured independently. On the other hand, if the observed spectrum of the protein mixture significantly deviates from the theoretical sum, then the overall protein conformation should have altered due to the interaction of two binding proteins. To determine whether the observed increase in secondary structural elements in the mixture of two proteins results in increased structure formation, we compared the theoretical sum (based on the sum of each data point arising from each individual) to the experimental CD spectra collected from a mixture of JDP2 and GR500 (Figure 2B). A comparison of the spectra from observed vs. theoretical sums showed a substantial increase in negative ellipticity at 222 nm in case of the experimental spectrum, compared with the theoretical sum of the individual spectra of JDP2 and GR500 (Figure 2B), suggesting that the observed increase in secondary structural elements in the complex are not additive, but due to the complex formation. Since JDP2 is a well structured bZIP protein (as judged by the spectrum in Figure 2A; and 32), our CD data strongly suggest that the increased structural elements must be coming primarily from GR500. A qualitative analysis of secondary structural elements using K2d algorithm [39] from CD data showed a significantly higher α-helical content in GR500 when bound to JDP2 in comparison to unbound GR500. This increased helical content in JDP2 bound GR500 appears to be coming at the expense of mostly random coil configuration. Since JDP2 binds to the core DBD and CTE, we further tested whether these observed changes in GR500 are happening in the DBD or the NTD. In spite of the fact that the DBD-CTE is sufficient to bind JDP2, as does GR500, our CD analyses under similar conditions showed that there was no significant structural change observed when the DBD (398–500 a.a.) was mixed with JDP2 (Figure 3A,B). It has earlier been reported that TFE (50%, vol/vol) had no effect on the CD spectra of JDP2, indicating that it is maximally folded in aqueous solution [32]. In contrast, the GR500, or isolated AF1, is reported to exhibit a substantial shift in conformation to more helical content in TFE [40]. These earlier published data further support our interpretations and validate our findings that binding of JDP2 to GR DBD leads to imposition of a compact structure in the otherwise ID AF1/NTD. To test for non-specific protein interactions, we recorded the CD spectra of an NTD/AF1 and JDP2 protein mixture (1∶2 molar ratios). As expected, there were no significant changes observed in the NTD/AF1 with JDP2 in the absence of DBD/CTE binding site (Figure 3C,D), suggesting that structural changes observed in GR500 are due to specific binding of JDP2 to its DBD/CTE. These results confirm that the increased structural elements observed in NTD/AF1 in the presence of JDP2 are not due to a mere presence of another protein in the mixture, but occurs as a result of JDP2 binding. Taken together, these results support the conclusion that binding of JDP2 to GR’s DBD causes the ID NTD/AF1 domain of GR to fold into a more compact structure. In the present study, JDP2 binding-induced structural changes in the GR NTD/AF1 domain represents a new feature of intra- and inter- molecular signaling leading to imposition of secondary/tertiary in the ID NTD/AF1 domain.

### JDP2-induced structure formation in the GR AF1 facilitates its interaction with specific transcriptional activator proteins

**Figure 4:**
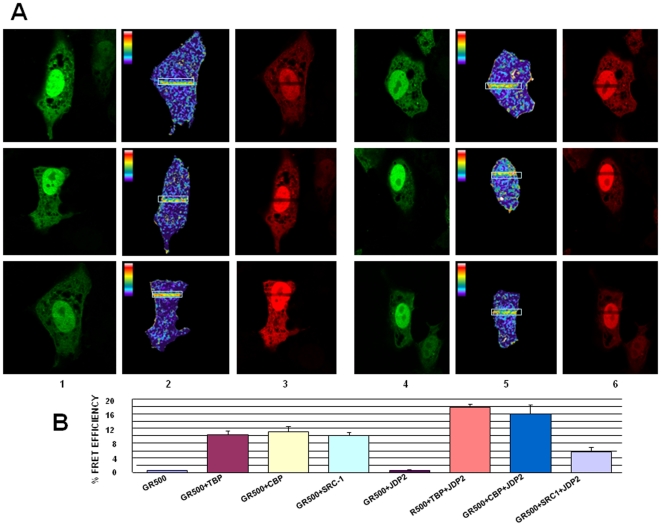
JDP2:DBD binding-induced conformational changes facilitate interactions of AF1 with specific coregulatory proteins in cell as assessed by FRET analyses. A) Representative same-cell images in the donor (CFP-GR500) and YFP-TBP, YFP-CBP, or YFP-SRC-1 channel before and after PB. The areas within the white boxes were photo bleached. 1 and 4 =  CFP- Pre PB; 2 and 5 =  CFP- Post PB; 3 and 6 =  YFP- Post PB. Upper panel, CFP-GR500 and YFP-TBP (left hand); and CFP-GR500 and YFP-TBP in the presence of JDP2 (right hand). Middle panel, CFP-GR500 and YFP-CBP (left hand); and CFP-GR500 and YFP-CBP in the presence of JDP2 (right hand). Lower panel, CFP-GR500 and YFP-SRC-1 (left hand); and CFP-GR500 and YFP-SRC-1 in the presence of JDP2 (right hand). Cells were also cotransfected with a promoter-reporter construct, GRE-SEAP (as described in Materials and Methods). B) Panel displays calculated average FRET efficiencies for each condition. Experiments were carried out three independent times and were analyzed and calculated average FRET efficiencies ± SD of 15 cells were graphed for each of the conditions.

It is presumed that AF1 makes physical interactions with other factors in order to transactivate gene(s) and that conditional folding is important for these interactions [17], [41]. We therefore evaluated whether the conformation induced in the ID AF1 domain due to JDP2 binding is important for specific protein-protein interactions. We applied the fluorescence resonance energy transfer (FRET) method. Plasmids expressing cyan fluorescent protein (CFP) and yellow fluorescent protein (YFP) were obtained, and from that we generated constructs that express the fluorophores linked to GR500 (CFP-GR500), TBP (YFP-TBP), CBP (YFP-CBP), or SRC-1 (YFP-SRC-1). The GR500 construct is constitutively active as a transcription factor while avoiding the possibility of any contribution from AF2 [21]. The constructs were co-transfected into GR-deficient CV-1 cells. Several control experiments were included to test for FRET as negative or positive controls. Our results show that GR500 interacts directly with TBP (Figure 4A; left hand, upper panel), CBP (Figure 4A; left hand, middle panel), or SRC-1 (Figure 4A; left hand, lower panel) in the nuclei of GR-deficient CV-1 cells co-transfected with GR500 ± each co-regulator. Interaction of AF1 with TBP (Figure 4A; right hand, upper panel) or CBP (Figure 4A; right hand, middle panel) is greatly enhanced when cells were co-transfected along with plasmid expressing JDP2. However, co-transfection of JDP2 failed to produce any significant change in the FRET efficiency between the GR AF1 and SRC-1 under similar conditions (Figure 4A; right hand, lower panel). A quantitative analysis of FRET data for each set of experiments is shown in Figure 4B. These results suggest that JDP2-induced conformational changes in ID NTD/AF1 allow its protein surfaces to facilitate AF1’s interaction with specific co-regulatory proteins, an essential requirement for activation domains of SHRs to regulate transcriptional activity of target gene [17].

### Effects of JDP2 on TBP, SRC-1 or CBP mediated enhancement of AF1-driven transcription

**Figure 5:**
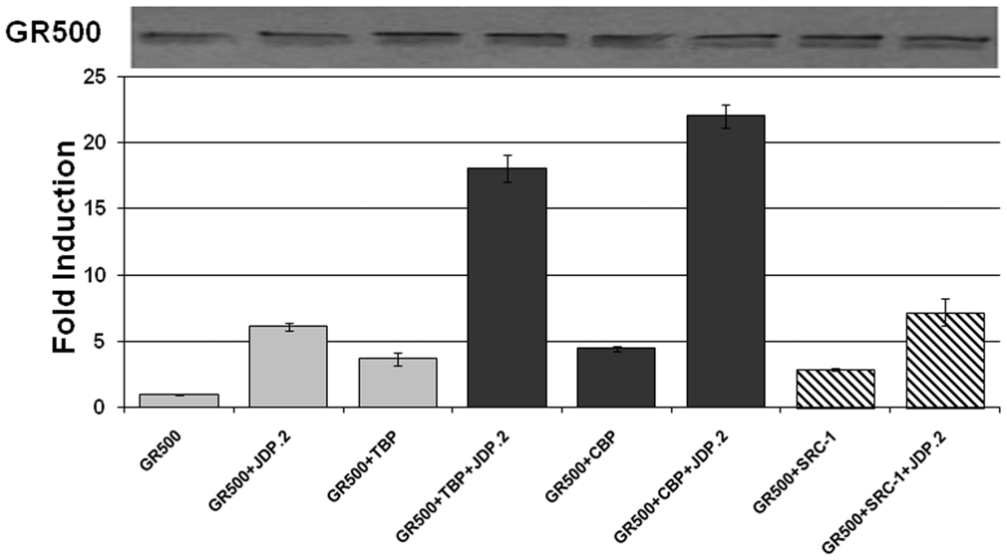
JDP2:DBD interaction-dependent cofactor-binding increases AF1-mediated transcriptional activity of a promoter containing 3xGRE as assessed by SEAP-based promoter:reporter assay. CV-1 cells cotransfected with vectors containing genes for GR500 with or without other cofactors (as indicated). Results are expressed as means ±SE. Experiments were repeated at least three times. Levels of significance were evaluated by a two-tailed paired Student's t test and P<0.05 was considered significant. Graphs were normalized to transfection efficiency of each construct assayed by immunoblot with specific antibody to GR (lower panel).

We tested the effects of JDP2-induced folding/binding events on AF1-driven transcription using GR-responsive promoters, in transient transfection-based reporter assays in GR-deficient CV-1 cells. The promoter-reporter plasmid (GRE-SEAP) contains three GREs upstream from a TATA-box and a reporter gene that encodes alkaline phosphatase secreted into the medium. To test the effects of these coregulators on transcription driven by human GR AF1, we co-transfected CV-1 cells with a GRE-dependent reporter gene, and constant amount of GR500 expression vector alone or with added vectors expressing TBP, SRC-1 or CBP. Lacking the LBD, GR500 is transcriptionally active without steroid and can induce genes and/or apoptosis in cells to nearly the same extent as steroid-bound holo-GR [21]. GR500 alone significantly increased reporter activity compared to empty vector alone, and input of the plasmids expressing TBP, SRC-1 or CBP gene enhanced the GR500 induction of the GRE–SEAP reporter several fold (Figure 5). These reporter activities were significantly enhanced when the plasmid expressing JDP2 was added (Figure 5). However, in the case of SRC-1, under similar conditions, we did not observe any significant increase compared to other co-regulators (TBP or CBP). The level of expression of GR500 was assessed using GR antibody and found to be equivalent. The western blot on the top of Figure 5 shows the level of GR500 expression in each case, and data represented are corrected to the efficiency of transfections. These results strongly suggest that the enhancement of GR-induced transcription by TBP, SRC-1 or CBP can be achieved through the AF1 region, and that JDP2 plays a role by inducing a functionally active compact structure that facilitates binding of selective coactivators to AF1 region. Enhanced GRE-mediated AF1 activity by JDP2 alone further confirms that JDP2 acts as a coregulator for the GR.

## Discussion

To promote molecular recognition with their physiological binding partners, eukaryotic genomes contain a number of proteins with ID regions/domains that are involved in signaling and regulation in nucleated cells [42], [43], [44], [45], [46], [47], [48], [49], [50]. Due to conformational flexibility, the ID AF1/NTD of the SHRs can include or exclude a variety of binding partners in a rapid and well coordinated manner for efficient and target specific regulation of gene expression [17]. Identification of a DBD-binding protein, JDP2 as a coregulator for GR AF1 activity represents a novel mode of regulation of AF1 activity. Since JDP2 was previously known to mediate the PR’s AF1 activity through similar mechanisms [31], [32], [33], [34], [35], it suggests that JDP2 mediated-AF1 activity may be a common mechanism for all members of the SHR family. In fact, JDP2 binding is mapped to the same region of GR and PR requiring the 2^nd^ zinc finger of the core DBD and the CTE [33]. Interestingly, the CTE is a short ID region that is not conserved in amino acids sequence among SHRs and is capable of binding both proteins and DNA. Studies have shown the CTE can adopt different structures and conformation dependent upon whether it is bound to DNA or free in solution.

It has previously been shown that JDP2 and DNA binding had distinct effects on conformation and folding of the hinge and NTD regions of PR as assessed by partial proteolysis [32]. In the earlier studies on JDP2 interaction with PR, JDP2 had no influence on PR binding to its response element DNA by gel mobility shift assays *in vitro* and a progesterone stimulated, PR-dependent recruitment of JDP2 by ChIP assay to the MMTV promoter in breast cancer cells was observed [31]. These data suggest that the effect of JDP2 on transcriptional activity of PR is not due to enhancing its binding to DNA. It has also been shown that common residues in the PR CTE (R637/K638) that interact with the minor groove of DNA and are required for binding and functional response to JDP2 [33]–[34]. Previous crystallography and NMR studies with PR showed that CTE is in a different conformation when bound to DNA or JDP2 [33], [34]. Since the CTE itself appears to be an ID region suggests the possibility that CTE interactions are involved in mediating allosteric coupling with the NTD through degenerate thermodynamic interactions between domains of SHRs as proposed by Hilser and Thompson [51]. From these data, it can be concluded that the ID CTE interacts transiently with DNA or JDP2 through interconverting conformers. Our FRET and promoter-reporter data (Figures 4 and 5) suggest that JDP2 binding does not interfere with the binding of GR500 with cognate DNA response element and the effect of JDP2 on transcriptional activity of GR is not due to enhancing its binding to DNA response element.

In many cases, the unfolded or partially folded regions of proteins take full shape when the protein interacts with its proper binding partner(s), that is, the molecules to which it must bind to carry out its function [48], [50]. Applied to the GR, this induced fit model of folding hypothesizes that AF1 is not fully structured *in vivo* until it binds one or another key partner molecule [17], [52]. We hypothesize that an induced conformation, or limited set of conformations, occurs in AF1 in order for it to carry out its transcription function. In this version of the model the proximity of the two proteins leads to rapid acquisition of functional structure in AF1. In this study we show that complex formation between the GR’s DBD/CTE and JDP2 is accompanied by changes in protein conformation in the NTD/AF1 domain. The most likely cause of this effect is by the highly structured JDP2 passing a signal through GR’s DBD to induce a folding event in the ID of the AF1/NTD. These conditional structural changes in the N-terminal region of the GR following JDP2 binding via the DBD may play an important role in triggering gene regulation. Assuming that these changes are taking place in AF1, it can be imagined that binding to JDP2 is required to bring the receptor's major transactivation domain into a conformation suited for its interaction with specific coactivators and/or proteins of the transcriptional machinery. Of course, this model in no way rules out the possibility of further structural changes in AF1, or the entire GR, as a result of those protein-protein interactions.

Our structural data from CD and proteolytic digestion experiments clearly show that binding with JDP2 leads to imposition of greater structure in the NTD/AF1 domain. Thus, the binding of JDP2 to GR’s DBD/CTE is not a simple tethering of the two molecules, but is an important step towards giving a folded functional structure to the AF1 domain through inherent inter-domain influences. Beyond this inherent structural effect, we have also shown that binding of the GR DBD to its cognate DNA response element causes structure to form in the GR NTD/AF1 [35]. Others have shown DNA binding effects on NTD/AF1 structure of the PR [53]. It has been proposed that DNA sequence of the various response elements found in specific genes may influence the fold and therefore the specific actions of AF1 [54]. If DBD:JDP2 interaction causes AF1 to assume a native, functional structure, then interaction of the AF1 domain with specific binding partner proteins should be enhanced. It has been shown that AF1 can bind to several proteins important for transcription [55], [56], [57]. Indeed, JDP2 binding-induced structural changes in NTD/AF1 appeared to be specific, since JDP2 enhanced AF1’s interaction with TBP and CBP, but failed to do so as efficiently for SRC-1. Further, JDP2 folding/binding events correlate well with AF1 activity. This is consistent with our hypothesis that JDP2-induced folding of the GR AF1 domain facilitates its interaction with specific coregulators.

We have earlier shown that in the presence of a naturally occurring osmolyte, trehalose, which can fold AF1 into functionally active conformation, facilitates AF1’s interaction with SRC-1 [58]. Therefore, it is logical to speculate that under physiological conditions not all events that are capable of folding AF1 open the same surfaces for its interactions with other binding partner proteins. It is possible that, JDP2-induced conformational changes may exclude certain proteins from the complex. Our earlier studies have shown that direct binding of TBP to AF1 and site-specific phosphorylation of AF1 result in induced structure formation in AF1 [25], [59]. It will be interesting to determine whether structural changes observed in AF1 under different physiological conditions are similar or unique. We hypothesize that a combination of various events, which may be cell- and promoter- specific, are needed to induce fully folded conformation in the AF1.

**Figure 6:**
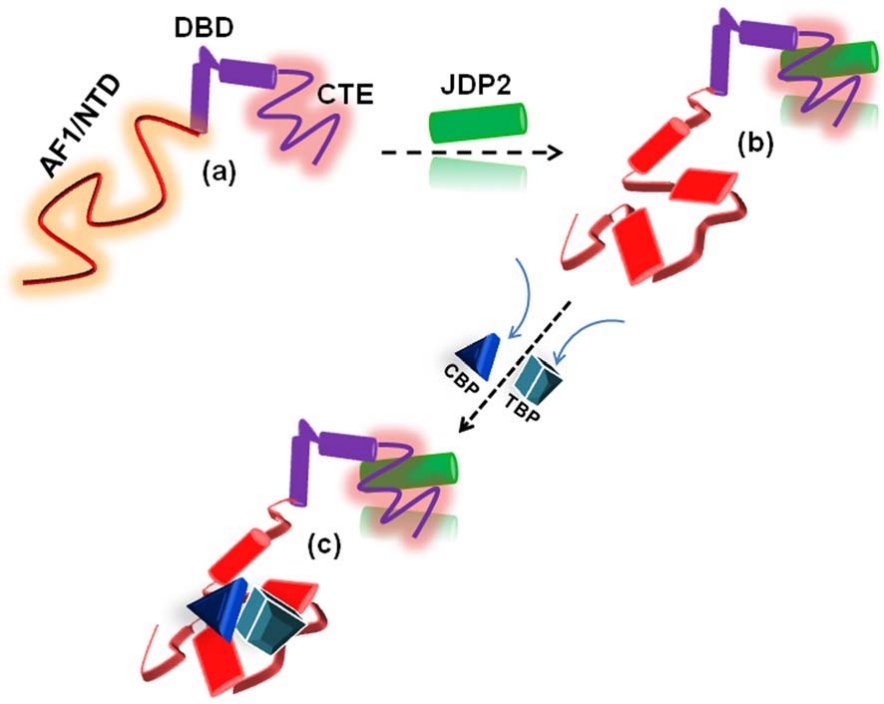
Proposed mechanism of effects of JDP2 binding/folding in the stimulation of GR’s AF1 activity. Compared to the highly structured GR’s DBD and LBD, the NTD/AF1 is mostly unstructured in solution (a). JDP2 interaction with the DBD/CTE transmits inter-domain signals to the AF1/NTD, resulting into secondary/tertiary structure formation in it (b). This induced structure in the AF1/NTD creates interaction surfaces for other coactivators (e.g., TBP and CBP) that mediate transcriptional activity of the AF1 (c). Undergoing conformational changes are indicated by different shapes and colors.

In sum, our data suggest that the assembly of GR:binding partner complex is an essential step in promoting AF1’s properly folded, functioning structure. Further, proteins that affect AF1 structure are not confined to those that bind directly to AF1 rather they can influence AF1 conformation through binding other domains of GR. The effect of JDP2 appears to be dependent on a specific inter-domain communication between the DBD and NTD. These data suggest that the transcriptional activity of the N-terminal domain involves protein folding that can be integrated allosterically through cofactor binding to the DBD. Also, whether specific GRE sequences are required for these effects remains to be seen. Overall, it appears that in the context of full length receptor under physiological conditions, AF1 adopts a set of conformations due to inter- and intra- molecular communications including but not limited to cofactor binding. These structurally modified AF1 conformations, and by large the entire NTD under specific conditions, may dictate the final outcome of the receptor:cofactor assembly leading to regulation of target genes (Figure 6). Physical interactions between the AF1 and AF2 may also influence these results.
